# From Rényi Entropy Power to Information Scan of Quantum States

**DOI:** 10.3390/e23030334

**Published:** 2021-03-12

**Authors:** Petr Jizba, Jacob Dunningham, Martin Prokš

**Affiliations:** 1 FNSPE, Czech Technical University in Prague, Břehová 7, 115 19 Praha 1, Czech Republic; proksma6@fjfi.cvut.cz; 2 Department of Physics and Astronomy, University of Sussex, Brighton BN1 9QH, UK; J.Dunningham@sussex.ac.uk

**Keywords:** Rényi entropy, Tsallis entropy, entropic uncertainty relations, quantum metrology

## Abstract

In this paper, we generalize the notion of Shannon’s entropy power to the Rényi-entropy setting. With this, we propose generalizations of the de Bruijn identity, isoperimetric inequality, or Stam inequality. This framework not only allows for finding new estimation inequalities, but it also provides a convenient technical framework for the derivation of a one-parameter family of Rényi-entropy-power-based quantum-mechanical uncertainty relations. To illustrate the usefulness of the Rényi entropy power obtained, we show how the information probability distribution associated with a quantum state can be reconstructed in a process that is akin to quantum-state tomography. We illustrate the inner workings of this with the so-called “cat states”, which are of fundamental interest and practical use in schemes such as quantum metrology. Salient issues, including the extension of the notion of entropy power to Tsallis entropy and ensuing implications in estimation theory, are also briefly discussed.

## 1. Introduction

The notion of entropy is undoubtedly one of the most important concepts in modern science. Very few other concepts can compete with it in respect to the number of attempts to clarify its theoretical and philosophical meaning [1]. Originally, the notion of entropy stemmed from thermodynamics, where it was developed to quantify the annoying inefficiency of steam engines. It then transmuted into a description of the amount of disorder or complexity in physical systems. Though many such attempts were initially closely connected with the statistical interpretation of the phenomenon of heat, in the course of time, they expanded their scope far beyond their original incentives. Along those lines, several approaches have been developed in attempts to quantify and qualify the entropy paradigm. These have been formulated largely independently and with different applications and goals in mind. For instance, in *statistical physics*, entropy counts the number of distinct microstates compatible with a given macrostate [2], in *mathematical statistics*, it corresponds to the inference functional for an updating procedure [3], and in *information theory*, it determines a limit on the shortest attainable encoding scheme [2,4].

Particularly distinct among these are the information-theoretic entropies (ITEs). This is not only because they discern themselves through their firm operational prescriptions in terms of coding theorems and communication protocols [5,6,7,8,9], but because they also offer an intuitive measure of disorder phrased in terms of missing information about a system. Apart from innate issues in communication theory, ITEs have also proved to be indispensable tools in other branches of science. Typical examples are provided by chaotic dynamical systems and multifractals (see, e.g., [10] and citations therein). Fully developed turbulence, earthquake analysis, and generalized dimensions of strange attractors provide further examples [11]. An especially important arena for ITEs in the past two decades has been quantum mechanics (QM) with applications ranging from quantum estimation and coding theory to quantum entanglement. The catalyst has been an infusion of new ideas from (quantum) information theory [12,13,14,15], functional analysis [16,17], condensed matter theory [18,19], and cosmology [20,21]. On the experimental front, the use of ITEs has been stimulated not only by new high-precision instrumentation [22,23] but also by, e.g., recent advances in stochastic thermodynamics [24,25] or observed violations of Heisenberg’s error-disturbance uncertainty relations [26,27,28,29,30].

In his seminal 1948 paper, Shannon laid down the foundations of modern information theory [5]. He was also instrumental in pointing out that, in contrast with discrete signals or messages where information is quantified by (Shannon’s) entropy, the cases with continuous variables are less satisfactory. The continuous version of Shannon’s entropy (SE)— the so-called differential entropy, may take negative values [5,31], and so it does not have the same status as its discrete-variable counterpart. To solve a number of information-theoretic problems related to continuous cases Shannon shifted the emphasis from the differential entropy to yet another object—entropy power (EP). The EP describes the variance of a would-be Gaussian random variable with the same differential entropy as the random variable under investigation. EP was used by Shannon [5,6] to bound the capacity of non-Gaussian additive noise channels. Since then, the EP has proved to be essential in a number of applications ranging from interference channels to secrecy capacity [32,33,34,35,36]. It has also led to new advances in information parametric statistics [37,38] and network information theory [39]. Apart from its significant role in information theory, the EP has found wide use in pure mathematics, namely in the theory of inequalities [39] and mathematical statistics and estimation theory [40].

Recent developments in information theory [41], quantum theory [42,43], and complex dynamical systems in particular [10,44,45] have brought about the need for a further extension of the concept of ITE beyond Shannon’s conventional type. Consequently, numerous generalizations have started to proliferate in the literature ranging from additive entropies [31,46] through a rich class of non-additive entropies [47,48,49,50,51,52] to more exotic types of entropies [53]. Particularly prominent among such generalizations are ITEs of Rényi and Tsallis, which both belong to a broader class of so-called Uffink entropic functionals [54,55]. Both Rényi entropy (RE) and Tsalli entropy (TE) represent one-parameter families of deformations of Shannon’s entropy. An important point related to the RE is that the RE is not just a theoretical construct, but it has a firm operational meaning in terms of various coding theorems [8,9]. Consequently, REs, along with their associated Rényi entropy powers (REPs), are, in principle, experimentally accessible [8,56,57]. That is indeed the case in specific quantum protocols [58,59,60]. In addition, REPs of various orders are often used as convenient measures of entanglement—e.g., REP of order 2, i.e., $N_{2}$ represents *tangle* τ (with $\sqrt{\tau }$ being *concurrence*) [61], $N_{1/2}$ is related to both *fidelity* *F* and *robustness* *R* of a pure state [62], $N_{\infty }$ quantifies the Bures distance to the closest separable pure state [63], etc. Even though our main focus here will be on REs and REPs since they are more pertinent in information theory, we will include some discussion related to Tsallis entropy powers at the end of this paper.

The aim of this paper is twofold. First, we wish to appropriately extend the notion of SE-based EP to the RE setting. In contrast to our earlier works on the topic [13,64], we will do it now by framing REP in the context of RE-based estimation theory. This will be done by judiciously generalizing such key notions as the De Bruijn identity, isoperimetric inequality (and ensuing Cramér–Rao inequality), and Stam inequality. In contrast to other similar works on the subject [65,66,67,68], our approach is distinct in three key respects: (a) we consistently use the notion of escort distribution and escort score vector in setting up the generalized De Bruijn identity and Fisher information matrix, (b) we generalize Stam’s uncertainty principle, and (c) Rényi EP is related to variance of the reference Gaussian distribution rather than the Rényi maximizing distribution. As a byproduct, we derive within such a generalized estimation theory framework the Rényi-EP-based quantum uncertainty relations (REPUR) of Schrödinger–Roberston type. The REPUR obtained coincides with our earlier result [13] that was obtained in a very different context by means of the Beckner–Babenko theorem. This in turn serves as a consistency check of the proposed generalized estimation theory. Second, we identify interesting new playgrounds for the Rényi EPs obtained. In particular, we asked ourselves a question: assuming one is able in specific quantum protocols to measure Rényi EPs of various orders, how does this constrain the underlying quantum state distribution? To answer this question, we invoke the concept of the *information distribution* associated with a given quantum state. The latter contains a complete “information scan” of the underlying state distribution. We set up a reconstruction method based on Hausdorff’s moment problem [69] to show explicitly how the information probability distribution associated with a given quantum state can be numerically reconstructed from EPs. This is a process that is analogous to quantum-state tomography. However, whereas tomography extracts the full density matrix from an ensemble using many measurements on a tomographically complete basis, the EP reconstruction method extracts the probability density on a given basis. This is an alternative approach that may be advantageous, for example, in quantum metrology schemes, where only knowledge of the local probability density rather than the full quantum state is needed [70].

The paper is structured as follows. In Section 2, we introduce the concept of Rényi’s EP. With quantum metrology applications in mind, we discuss this in the framework of estimation theory. First, we duly generalize the notion of Fisher information (FI) by using a Rényi entropy version of De Bruijn’s identity. In this connection, we emphasize the role of the so-called *escort distribution*, which appears naturally in the definition of higher-order *score functions*. Second, we prove the RE-based isoperimetric inequality and ensuing Cramér–Rao inequality and find how the knowledge of Fisher information matrix restricts possible values of Rényi’s EP. Finally, we further illuminate the role of Rényi’s EP by deriving (through the Stam inequality) Rényi’s EP-based quantum uncertainty relations for conjugate observables. To flesh this out, the second part of the paper is devoted to the development of the use of Rényi EPs to extract the quantum state from incomplete data. This is of particular interest in various quantum metrology protocols. To this end, we introduce in Section 3 the concepts of information distribution, and, in Section 4, we show how cumulants of the information distribution can be obtained from knowledge of the EPs. With the cumulants at hand, one can reconstruct the underlying information distribution in a process which we call an *information scan*. Details of how one could explicitly realize such an information scan for quantum state PDFs are provided in Section 5. There we employ generalized versions of Gram–Charlier A and the Edgeworth expansion. In Section 6, we illustrate the inner workings of the information scan using the example of a so-called *cat state*. This state is of interest in applications of quantum physics such as quantum-enhanced metrology, which is concerned with the optimal extraction of information from measurements subject to quantum mechanical effects. The cat state we consider is a superposition of the vacuum state and a coherent state of the electromagnetic field; two cases are studied comprising different probabilistic weightings of the superposition state corresponding to *balanced* and *unbalanced* cat states. Section 7 is dedicated to EPs based on Tsallis entropy. In particular, we show that Rényi and Tsallis EPs coincide with each other. This, in turn, allows us to phrase various estimation theory inequalities in terms of TE. In Section 7, we end with conclusions. For the reader’s convenience, we relegate some technical issues concerning the generalized De Bruijn identity and associated isoperimetric and Stam inequalities to three appendices.

## 2. Rényi Entropy Based Estimation Theory and Rényi Entropy Powers

In this section, we introduce the concept of Rényi’s EP. With quantum metrology applications in mind, we discuss this in the framework of estimation theory. This will not only allow us to find new estimation inequalities, such as the Rényi-entropy-based De Bruijn identity, isoperimetric inequality, or Stam inequality, but it will also provide a convenient technical and conceptual frame for deriving a one-parameter family of Rényi-entropy-power-based quantum-mechanical uncertainty relations.

### 2.1. Fisher Information—Shannon’s Entropy Approach

First, we recall that the Fisher information matrix J(X) of a random vector $\left\{X_{i}\right\}$ in $R^{D}$ with the PDF F(x) is defined as [38]

(1) $$\begin{array}{c} J(X)\;=\;\mathrm{cov}(V(X))\;, \end{array}$$

where the covariance matrix is associated with the random zero-mean vector—the so-called *score vector*, as

(2) $$\begin{array}{c} V(x)\;=\;\nabla F(x)/F(x)\;. \end{array}$$

A corresponding trace of J(X), i.e.,

(3) $$\begin{array}{c} J\left(X\right)\;=\;\mathrm{Tr}\left(J\left(X\right)\right)\;=\;\mathrm{var}\left(V\left(X\right)\right)\;=\;E\left(V^{2}\left(X\right)\right)\;, \end{array}$$

is known as the Fisher information. Both the FI and FI matrix can be conveniently related to Shannon’s differential entropy via De Bruijn’s identity [66,67].

*De Bruijn’s identity:* Let $\left\{X_{i}\right\}$ be a random vector in $R^{D}$ with the PDF F(x) and let $\left\{Z_{i}^{{}_{G}}\right\}$ be a Gaussian random vector (noise vector) with zero mean and unit-covariance matrix, independent of $\left\{X_{i}\right\}$. Then,

(4) $$\begin{array}{c} \frac{d}{d\epsilon }H\left(X+\sqrt{\epsilon }\;\;Z^{{}_{G}}\right){|}_{\epsilon =0}\;=\;\frac{1}{2}J\left(X\right)\;, \end{array}$$

where

(5) $$\begin{array}{c} H\left(X\right)\;=\;-\int_{R^{D}}F\left(x\right)\log F\left(x\right)\;dx\;, \end{array}$$

is Shannon’s differential entropy (measured in *nats*). In the case when the independent additive noise $\left\{Z_{i}\right\}$ is non-Gaussian with zero mean and covariance matrix Σ=cov(Z), then the following generalization holds [67]:

(6) $$\begin{array}{c} \frac{d}{d\epsilon }H\left(X+\sqrt{\epsilon }\;\;Z\right){|}_{\epsilon =0}\;=\;\frac{1}{2}\mathrm{Tr}\left(J(X)\Sigma \right). \end{array}$$

The key point about De Bruijn’s identity is that it provides a very useful intuitive interpretation of FI, namely, FI quantifies the sensitivity of transmitted (Shannon type) information to an arbitrary independent additive noise. An important aspect that should be stressed in this context is that FI as a quantifier of sensitivity depends only on the covariance of the noise vector, and thus it is independent of the shape of the noise distribution. This is because De Bruijn’s identity remains unchanged for both Gaussian and non-Gaussian additive noise with the same covariance matrix.

### 2.2. Fisher Information—Rényi’s Entropy Approach

We now extend the notion of the FI matrix to the Rényi entropy setting. A natural way to do it is via an extension of De Bruijn’s identity to Rényi entropies. In particular, the following statement holds:

*Generalized De Bruijn’s identity:* Let $\left\{X_{i}\right\}$ be a random vector in $R^{D}$ with the PDF F(x) and let $\left\{Z_{i}\right\}$ be an independent (generally non-Gaussian) noise vector with the zero mean and covariance matrix Σ=cov(Z), then, for any q>0

(7) $$\begin{array}{c} \frac{d}{d\epsilon }I_{q}\left(X+\sqrt{\epsilon }\;\;Z\right){|}_{\epsilon =0}\;=\;\frac{1}{2q}\mathrm{Tr}\left(J_{q}\left(X\right)\Sigma \right)\;, \end{array}$$

where

(8) $$\begin{array}{c} I_{q}\;=\;\frac{1}{1-q}\log\int_{R^{D}}F^{q}\left(x\right)dx\;,\;\;\;\;q>0\;, \end{array}$$

is *Rényi’s differential entropy* (measured in *nats*) with $I_{1}=H$. The ensuing FI matrix of order *q* has the explicit form

(9) $$\begin{array}{c} J_{q}\left(X\right)\;=\;{\mathrm{cov}}_{q}\left(V_{q}\left(X\right)\right)\;, \end{array}$$

with the score vector

(10) $$\begin{array}{c} V_{q}\left(x\right)\;=\;\nabla \rho_{q}\left(x\right)/\rho_{q}\left(x\right)\;=\;q\nabla F\left(x\right)/F\left(x\right)\;=\;qV\left(x\right)\;. \end{array}$$

Here, $\rho_{q}=F^{q}/\int_{R^{D}}F^{q}dx$ is the so-called *escort distribution* [71]. The “${\mathrm{cov}}_{q}$” denotes the covariance matrix computed with respect to $\rho_{q}$. Proofs of both the conventional (i.e., Shannon entropy based) and generalized (i.e., Rényi entropy based) De Bruijn’s identity are provided in Appendix A. There we also discuss some further useful generalizations of De Bruijn’s identity. Finally, as in the Shannon case, we define the FI of order *q*—denoted as $J_{q}\left(X\right)$, as

(11) $$\begin{array}{c} \mathrm{Tr}\left(J_{q}\left(X\right)\right)\;\equiv \;J_{q}\left(X\right)\;. \end{array}$$

### 2.3. Rényi’s Entropy Power and Generalized Isoperimetric Inequality

Similarly as in conventional estimation theory, one can expect that there should exist a close connection between the FI matrix $J_{q}\left(X\right)$ and the corresponding Rényi entropy power $N_{p}\left(X\right)$. In Shannon’s information theory, such a connection is phrased in terms of isoperimetric inequality [67]. Here, we prove that a similar relationship works also in Rényi’s information theory.

Let us start by introducing the concept of Rényi’s entropy power. This is defined as the solution of the equation [13,64]

(12) $$\begin{array}{c} I_{p}\left(X\right)\;=\;I_{p}\left(\sqrt{N_{p}\left(X\right)}\cdot Z^{{}_{G}}\right)\;, \end{array}$$

where $\left\{Z_{i}^{{}_{G}}\right\}$ represents a Gaussian random vector with a zero mean and unit covariance matrix. Thus, $N_{p}\left(X\right)$ denotes the variance of a would be Gaussian distribution that has the same Rényi information content as the random vector $\left\{X_{i}\right\}$ described by the PDF F(x). Expression (12) was studied in [13,64,72], where it was shown that the only class of solutions of (12) is

(13) $$\begin{array}{c} N_{p}\left(X\right)\;=\;\frac{1}{2\pi }p^{-p^{\prime }/p}\exp\left(\frac{2}{D}\;\;I_{p}\left(X\right)\right), \end{array}$$

with $1/p+1/p^{\prime }=1$ and $p\in R^{+}$. In addition, when $p\to 1_{+}$, one has $N_{p}\left(X\right)\to N\left(X\right)$, where N(X) is the conventional Shannon entropy power [5]. In this latter case, one can use the *asymptotic equipartition property* [55,73] to identify N(X) with “typical size” of a state set, which in the present context is the effective support set size for a random vector. This, in turn, is equivalent to Einstein’s entropic principle [74]. In passing, it should be noted that the form of the Rényi EP expressed in (13) is not universally accepted version. In a number of works, it is defined merely as an exponent of RE, see, e.g., [75,76]. Our motivation for the form (13) is twofold: first, it has a clear interpretation in terms of variances of Gaussian distributions and, second, it leads to simpler formulas, cf. e.g., Equation (22).

*Generalized isoperimetric inequality:* Let $\left\{X_{i}\right\}$ be a random vector in $R^{D}$ with the PDF F(x). Then,

(14) $$\begin{array}{c} \frac{1}{D}N_{q}\left(X\right)J_{q}\left(X\right)\;\geq \;N_{q}\left(X\right){\left[\det\left(J_{q}\left(X\right)\right)\right]}^{1/D}\;\geq \;1\;, \end{array}$$

where the Rényi parameter q≥1. We relegate the proof of the generalized isoperimetric inequality to Appendix B.

It is also worth noting that the relation (14) implies another important inequality. By using the fact that the Shannon entropy is maximized (among all PDF’s with identical covariance matrix Σ) by the Gaussian distribution, we have $N_{1}\left(X\right)\leq \det{\left(\Sigma \right)}^{1/D}$ (see, e.g., [77]). If we further employ that $I_{q}$ is a monotonously decreasing function of *q*, see, e.g., [31,78], we can write (recall that q≥1)

(15) $$\begin{array}{c} \frac{q^{1/(q-1)}}{e}\;\;N_{q}\;\leq \;N_{1}\;=\;\frac{\exp(\frac{2}{D}I_{1})}{2\pi e}\;\leq \;\det{\left(\Sigma \right)}^{1/D}. \end{array}$$

The isoperimetric inequality (14) then implies

(16) $$\begin{array}{c} \det\left(\Sigma \left(X\right)\right)\;\geq \;\frac{{\left(q^{1/(q-1)}\right)}^{D}}{e^{D}\det\left(J_{q}\left(X\right)\right)}\;\geq \;\frac{1}{e^{D}\det\left(J_{q}\left(X\right)\right)}\;. \end{array}$$

We can further use the inequality

(17) $$\begin{array}{c} \frac{1}{D}\mathrm{Tr}\left(A\right)\;\geq \;{\left[\det\left(A\right)\right]}^{1/D}\;, \end{array}$$

(valid for any positive semi-definite D×D matrix A) to write

(18) $$\begin{array}{c} \sigma^{2}\left(X\right)\;=\;\frac{1}{D}\mathrm{Tr}\left(\Sigma \left(X\right)\right)\;=\;\frac{1}{D}\sum_{i=1}^{D}\mathrm{Var}\left(X_{i}\right)\;\geq \;\frac{Dq^{1/(q-1)}}{eJ_{q}\left(X\right)}\;\geq \;\frac{D}{eJ_{q}\left(X\right)}\;, \end{array}$$

where $\sigma^{2}$ is an average variance per component.

Relations (16)–(18) represent the *q*-generalizations of the celebrated Cramér–Rao information inequality. In the limit of q→1, we recover the standard Cramér–Rao inequality that is widely used in statistical inference theory [38,79]. A final logical step needed to complete the proof of REPURs is represented by the so-called generalized Stam inequality. To this end, we first define the concept of *conjugate random variables*. We say that random vectors $\left\{X_{i}\right\}$ and $\left\{Y_{i}\right\}$ in $R^{D}$ are conjugate if their respective PDF’s F(x) and G(y) can be written as

(19) $$\begin{array}{c} F\left(x\right)\;=\;|\varphi_{{}_{F}}{\left(x\right)|}^{2}/\left|\;\right|\varphi_{{}_{F}}{\left|\;\right|}_{2}^{2}\;,\;\;\;\;\;\;\;G\left(y\right)\;=\;|\varphi_{{}_{G}}{\left(y\right)|}^{2}/\left|\;\right|\varphi_{{}_{G}}{\left|\;\right|}_{2}^{2}\;, \end{array}$$

where the (generally complex) probability amplitudes $\varphi_{{}_{F}}\left(x\right)\in L_{2}\left(R^{D}\right)$ and $\varphi_{{}_{G}}\left(y\right)\in L_{2}\left(R^{D}\right)$ are mutual Fourier images, i.e.,

(20) $$\begin{array}{c} \varphi_{{}_{F}}\left(x\right)\;=\;{\hat{\varphi }}_{{}_{G}}\left(x\right)\;=\;\int_{R^{D}}e^{2\pi ix.y}\;\varphi_{{}_{G}}\left(y\right)\;dy\;, \end{array}$$

and analogously for $\varphi_{{}_{G}}\left(y\right)={\hat{\varphi }}_{{}_{F}}\left(y\right)$. With this, we can state the generalized Stam inequality.

*Generalized Stam inequality (Stam’s uncertainty principle):* Let $\left\{X_{i}\right\}$ and $\left\{Y_{i}\right\}$ be conjugate random vectors in $R^{D}$. Then,

(21) $$\begin{array}{c} 16\pi^{2}N_{q}\left(Y\right)\;\geq \;{\left[\det\left(J_{r}\left(X\right)\right)\right]}^{1/D}\;, \end{array}$$

is valid for any r∈[1,∞) and q∈[1/2,1] that are connected via the relation 1/r+1/q=2. In particular, if we define $r^{\prime }=2r$ and $q^{\prime }=2q$, then $r^{\prime }$ and $q^{\prime }$ are Hölder conjugates. A proof of the generalized Stam inequality is provided in Appendix C.

Let us now consider Hölder conjugate indices *p* and *q* with p∈[2,∞) (so that q∈[1,2]). Combining the isoperimetric inequality (14) together with the generalized Stam inequality (21), we obtain the following one-parameter class of REP-based inequalities

(22) $$\begin{array}{ccc} N_{p/2}\left(X\right)N_{q/2}\left(Y\right) & = & N_{p/2}\left(X\right)\frac{{\left[\det\left(J_{p/2}\left(X\right)\right)\right]}^{1/D}}{{\left[\det\left(J_{p/2}\left(X\right)\right)\right]}^{1/D}}N_{q/2}\left(Y\right)\\ & \geq & \frac{N_{q/2}\left(Y\right)}{{\left[\det\left(J_{p/2}\left(X\right)\right)\right]}^{1/D}}\;\geq \;\frac{1}{16\pi^{2}}\;. \end{array}$$

By symmetry, the role of *q* and *p* can be reversed. In Refs. [13,64], we presented an alternative derivation of inequalities (22) that was based on the Beckner–Babenko theorem. There it was also proved that the inequality saturates if and only if the distributions involved are Gaussian. The only exception to this rule is for the asymptotic values p=1 and q=∞ (or vice versa) where the saturation happens whenever the peak of F(x) and tail of G(y) (or vice versa) are Gaussian.

The passage to quantum mechanics is quite straightforward. First, we realize that, in QM, the Fourier conjugate wave functions are related via two reciprocal relations

(23) $$\begin{array}{c} \psi_{{}_{F}}\left(x\right)\;=\;\int_{R^{D}}e^{iy\cdot x/\hbar }\;\;\psi_{{}_{G}}\left(y\right)\;\;\frac{dy}{{\left(2\pi \hbar \right)}^{D/2}}\;,\\ \psi_{{}_{G}}\left(y\right)\;=\;\int_{R^{D}}e^{-iy\cdot x/\hbar }\;\;\psi_{{}_{F}}\left(x\right)\;\;\frac{dx}{{\left(2\pi \hbar \right)}^{D/2}}\;. \end{array}$$

The Plancherel (or Riesz–Fischer) equality implies that, when $\left|\;\right|\psi_{{}_{F}}{\left|\;\right|}_{2}=1$, then also automatically $\left|\;\right|\psi_{{}_{G}}{\left|\;\right|}_{2}=1$ (and vice versa). Thus, the connection between amplitudes $\varphi_{{}_{F}}$ and $\varphi_{{}_{G}}$ from (19) and amplitudes $\psi_{{}_{F}}$ and $\psi_{{}_{G}}$ from (23) is

(24) $$\begin{array}{c} \varphi_{{}_{F}}\left(x\right)\;=\;{\left(2\pi \hbar \right)}^{D/4}\psi_{{}_{F}}\left(\sqrt{2\pi \hbar }\;\;x\right)\;,\\ \varphi_{{}_{G}}\left(y\right)\;=\;{\left(2\pi \hbar \right)}^{D/4}\psi_{{}_{G}}\left(\sqrt{2\pi \hbar }\;\;y\right)\;. \end{array}$$

The factor ${\left(2\pi \hbar \right)}^{D/4}$ ensures that also $\varphi_{{}_{F}}$ and $\varphi_{{}_{G}}$ functions are normalized (in the sense of ${\left|\;|\ldots |\;\right|}_{2}$) to unity; however, due to Equation (19), it might be easily omitted. The corresponding Rényi EPs change according to

(25) $$\begin{array}{c} N_{p/2}\left(X\right)\;\equiv \;N_{p/2}\left(F\right)\;\;\mapsto \;\;N_{p/2}\left(\right|\psi_{{}_{F}}{|}^{2})\;=\;2\pi \hbar N_{p/2}\left(F\right)\;,\\ N_{q/2}\left(Y\right)\;\equiv \;N_{q/2}\left(G\right)\;\;\mapsto \;\;N_{q/2}\left(\right|\psi_{{}_{G}}{|}^{2})\;=\;2\pi \hbar N_{q/2}\left(G\right)\;, \end{array}$$

and hence REP-based inequalities (22) acquire in the QM setting a simple form

(26) $$\begin{array}{c} N_{p/2}\left({\left|\psi_{{}_{F}}\right|}^{2}\right)N_{q/2}\left(\right|\psi_{{}_{G}}{|}^{2})\;\geq \;\frac{\hbar^{2}}{4}\;. \end{array}$$

This represents an infinite tower of mutually distinct (generally irreducible) REPURs [13].

At this point, some comments are in order. First, historically, the most popular quantifier of quantum uncertainty has been *variance* because it is conceptually simple and relatively easily extractable from experimental data. The variance determines the measure of uncertainty in terms of the fluctuation (or spread) around the mean value, which, while useful for many distributions, does not provide a sensible measure of uncertainty in a number of important situations including multimodal [12,13,64] and heavy-tailed distributions [13,14,64]. To deal with this, a multitude of alternative (non-variance based) measures of uncertainty in quantum mechanics (QM) have emerged. Among these, a particularly prominent role is played by information entropies such as the Shannon entropy [63], Rényi entropy [63,64], Tsallis entropy [80], associated differential entropies, and their quantum-information generalizations [13,15,64]. REPURs (26) fit into this framework of entropic QM URs. In connection with (26), one might observe that the conventional URs based on variances—so-called Robertson–Schrödinger URs [81,82]) and Shannon differential entropy based URs (e.g., Hirschman or Białynicki–Birula URs [15,83]) naturally appear as special cases in this hierarchy. Second, the ITEs enter quantum information theory typically in three distinct ways: (a) as a measure of the quantum information content (e.g., how many qubits are needed to encode the message without loss of information), (b) as a measure of the classical information content (e.g., amount of information in bits that can be recovered from the quantum system) and (c) to quantify the entanglement of pure and mixed bipartite quantum states. Logarithms in base 2 are used because, in quantum information, one quantifies entropy in bits and qubits (rather than nats). This in turn also modifies Rényi’s EP as

(27) $$\begin{array}{c} \frac{1}{2\pi }p^{-p^{\prime }/p}e^{\left(\frac{2}{D}\;\;\cdots \right)}\;\;\mapsto \;\;\frac{1}{2\pi }p^{-p^{\prime }/p}\;\;2^{\left(\frac{2}{D}\;\;\cdots \right)}\;. \end{array}$$

In the following, we will employ this QM practice.

## 3. Information Distribution

To put more flesh on the concept of Rényi’s EP, we devote the rest of this paper to the development of the methodology and application of Rényi EPs in extracting quantum states from incomplete data. The technique of quantum tomography is widely used for this purpose and involves making many different measurements on an ensemble of identical copies of a quantum state with a tomographically complete measurement basis [84,85]. This process is very measurement-intensive, scaling exponentially with the number of particles and so methods have been developed to approximate it with fewer measurements [86].

However, the method of Rényi EPs provides an efficient alternative approach. Instead of reconstructing the full quantum state, this process extracts the PDF of the quantum state in a given basis. For a broad class of quantum metrology problems, local rather than global approaches are preferred [70] and, for these, the local PDF of the state at each sensor is needed rather than the full density matrix. With this in mind, we first start with the notion of the information distribution.

Let F(x) be the PDF for the random variable X. We define the *information random variable* $i_{X}\left(X\right)$ so that $i_{X}\left(x\right)=\log_{2}1/F\left(x\right)$. In other words, $i_{X}\left(x\right)$ represents the information in x with respect to F(x). In this connection, it is expedient to introduce the cumulative distribution function for $i_{X}\left(X\right)$ as

(28) $$\begin{array}{c} \;℘\left(y\right)=\int_{-\infty }^{y}d℘\left(i_{X}\right)=\int_{R^{D}}F\left(x\right)\theta \left(\log_{2}F\left(x\right)+y\right)dx\;. \end{array}$$

The function ℘(y) thus represents the probability that the random variable $i_{X}\left(X\right)$ is less than or equal to *y*. We have denoted the corresponding probability measure as $d℘(i_{X})$. Taking the Laplace transform of both sides of (28), we get

(29) $$\begin{array}{c} \;\;L\left\{℘\right\}\left(s\right)\;=\;\int_{R^{D}}\;\;\;F\left(x\right)\;\;\frac{e^{s\log_{2}F\left(x\right)}}{s}\;\;dx\;=\;\frac{E\left[e^{s\log_{2}F}\right]}{s}\;, \end{array}$$

where $E\left[\cdots \right]$ denotes the mean value with respect to F. By assuming that ℘(x) is smooth, then the PDF associated with $i_{X}\left(X\right)$—the so-called *information PDF*—is

(30) $$\begin{array}{c} g\left(y\right)\;=\;\frac{d℘(y)}{dy}\;=\;L^{-1}\left\{E\left[e^{s\log_{2}F}\right]\right\}\;\left(y\right)\;. \end{array}$$

Setting s=(p−1)log2, we have

(31) $$\begin{array}{c} L\left\{g\right\}\left(s=\left(p-1\right)\log2\right)\;=\;E\left[2^{\left(1-p\right)i_{X}}\right]. \end{array}$$

The mean here is taken with respect to the PDF *g*. Equation (31) can also be written explicitly as

(32) $$\begin{array}{c} \int_{R^{D}}dx\;\;F^{p}\left(x\right)\;=\;\int_{R}g\left(y\right)2^{(1-p)y}dy\;. \end{array}$$

Note that, when $F^{p}$ is integrable for p∈[1,2], then (32) ensures that the moment-generating function for g(x) PDF exists. Thus, in particular, the moment-generating function exists when F(x) represents Lévy α-stable distributions, including the heavy-tailed stable distributions (i.e, PDFs with the Lévy stability parameter α∈(0,2]). The same holds for $\hat{F}$ and $p^{\prime }\in \left[2,\infty \right)$ due to the Beckner–Babenko theorem [13,87,88].

## 4. Reconstruction Theorem

Since L{g}(s) is the *moment-generating function* of the random variable $i_{X}\left(X\right)$, one can generate all moments of the PDF g(x) (if they exist) by taking the derivatives of L{g} with respect to *s*. From a conceptual standpoint, it is often more useful to work with cumulants rather than moments. Using the fact that the *cumulant generating function* is simply the (natural) logarithm of the moment-generating function, we see from (32) that the differential RE is a reparametrized version of the cumulant generating function of the information random variable $i_{X}\left(X\right)$. In fact, from (31), we have

(33) $$I_{p}\left(X\right)\;=\;\frac{1}{\left(1-p\right)}\log_{2}E\left[2^{\left(1-p\right)i_{X}}\right]\;.$$

To understand the meaning of REPURs, we begin with the cumulant expansion (33), i.e.,

(34) $$\begin{array}{c} pI_{1-p}\left(X\right)\;=\;\log_{2}e\sum_{n=1}^{\infty }\frac{\kappa_{n}\left(X\right)}{n!}{\left(\frac{p}{\log_{2}e}\right)}^{n}\;, \end{array}$$

where $\kappa_{n}\left(X\right)\equiv \kappa_{n}\left(i_{X}\right)$ denotes the *n*-th cumulant of the information random variable $i_{X}\left(X\right)$ (in units of *bits*${}^{n}$). We note that

(35) $$\begin{array}{c} \kappa_{1}\left(X\right)\;=\;E\left[i_{X}\left(X\right)\right]\;=\;H\left(X\right)\;,\\ \kappa_{2}\left(X\right)\;=\;E\left[i_{X}{\left(X\right)}^{2}\right]-{\left(E\left[i_{X}\left(X\right)\right]\right)}^{2}\;, \end{array}$$

i.e., they represent the Shannon entropy and *varentropy*, respectively. By employing the identity

(36) $$\begin{array}{ccc} \;I_{1-p}\left(X\right) & = & \;\frac{D}{2}\log_{2}\left[2\pi {\left(1-p\right)}^{-1/p}N_{1-p}\left(X\right)\right]\;, \end{array}$$

we can rewrite (34) in the form

(37) $$\begin{array}{c} \log_{2}\left[N_{1-p}\left(X\right)\right]\;=\;\log_{2}\left[\frac{{\left(1-p\right)}^{1/p}}{2\pi }\right]\;+\;\frac{2}{D}\sum_{n=1}^{\infty }\frac{\kappa_{n}\left(X\right)}{n!}{\left(\frac{p}{\log_{2}e}\right)}^{n-1}\;\;. \end{array}$$

From (37), one can see that

(38) $$\begin{array}{ccc} \;\kappa_{n}\left(X\right)\; & = & \;{\left.\frac{nD}{2}{\left(\log_{2}e\right)}^{n-1}\frac{d^{n-1}\log_{2}\left[N_{1-p}\left(X\right)\right]}{dp^{n-1}}\right|}_{p=0}\\ & + & \;\frac{D}{2}{\left(\log_{2}e\right)}^{n}\left[\left(n-1\right)!+\delta_{1n}\log2\pi \right]\;, \end{array}$$

where $\delta_{1n}$ is the Kronecker delta function that has a value of one if n=1, or zero otherwise. In terms of the Grünwald–Letnikov derivative formula (GLDF) [89], we can rewrite (38) as

(39) κn(X)=limΔ→0nD2(log2e)nΔn−1∑k=0n−1(−1)kn−1klogN1+kΔ(X)+D2(log2e)n(n−1)!+δ1nlog2π.

Thus, in order to determine the first *m* cumulants of $i_{X}\left(X\right)$, we need to know all $N_{1},N_{1+\Delta },\ldots ,N_{1+(m-1)\Delta }$ entropy powers. In practice, Δ corresponds to a characteristic resolution scale for the entropy index which will be chosen appropriately for the task at hand, but is typically of the order $10^{-2}$. Note that the last term in (38) and (39) can be also written

(40) $$\begin{array}{c} \frac{D}{2}{\left(\log_{2}e\right)}^{n}\left[\left(n-1\right)!+\delta_{1n}\log2\pi \right]\;\;=\;\kappa_{n}\left(Z_{G}^{1\;I}\right)\;\equiv \;\kappa_{n}\left(i_{Y}\right)\;, \end{array}$$

with Y being the random variable distributed with respect to the *Gaussian* distribution $Z_{G}^{1\;I}$ with the *unit* covariance matrix.

When all the cumulants exist, then the problem of recovering the underlying PDF for $i_{X}\left(X\right)$ is equivalent to the *Stieltjes* moment problem [90]. Using this connection, there are a number of ways to proceed; the PDF in question can be reconstructed e.g., in terms of sums involving orthogonal polynomials (e.g., the Gram–Charlier A series or the Edgeworth series [91]), the inverse Mellin transform [92], or via various maximum entropy techniques [93]. Pertaining to this, the theorem of Marcinkiewicz [94] implies that there are no PDFs for which $\kappa_{m}=\kappa_{m+1}=\ldots =0$ for m≥3. In other words, the cumulant generating function cannot be a finite-order polynomial of degree greater than 2. The important exceptions, and indeed the only exceptions to Marcinkiewicz’s theorem are the *Gaussian* PDFs that can have the first two cumulants nontrivial and $\kappa_{3}=\kappa_{4}=\ldots =0$. Thus, apart from the special case of Gaussian PDFs where only $N_{1}$ and $N_{1+\Delta }$ are needed, one needs to work with as many entropy powers $N_{1+k\Delta },k\in N$ (or ensuing REPURs) as possible to receive as much information as possible about the structure of the underlying PDF. In theory, the whole infinite tower of REPURs would be required to uniquely specify a system’s information PDF. Note that, for *Gaussian* information PDFs, one needs only $N_{1}$ and $N_{1+\Delta }$ to reconstruct the PDF uniquely. From (37) and (39), we see that knowledge of $N_{1}$ corresponds to $\kappa_{1}\left(X\right)=H\left(X\right)$ while $N_{1+\Delta }$ further determines $\kappa_{2}$, i.e., the varentropy. Since $N_{1}$ is involved (via (39)) in the determination of all cumulants, it is the most important entropy power in the tower. Thus, the entropy powers of a given process have an equivalent meaning to the PDF: they describe the morphology of uncertainty of the observed phenomenon.

We should stress that the focus of the reconstruction theorem we present is on cumulants $\kappa_{n}$ which can be directly used for a shape estimation of g(x) but not F(x). However, by knowing g(y), we have a complete “information scan” of F(x). Such an information scan is, however, not unique, indeed, two PDFs that are rearrangements of each other—i.e., *equimeasurable* PDFs, have identical ℘(y) and g(y). Even though equimeasurable PDFs cannot be distinguished via their entropy powers, they can be, as a rule, distinguished via their respective momentum-space PDFs and associated entropy powers. Thus, the information scan has a tomographic flavor to it. From the multi-peak structure of g(y), one can determine the *number* and *height* of the stationary points. These are invariant characteristics of a given family of equimeasurable PDFs. This will be further illustrated in Section 6.

## 5. Information Scan of Quantum-State PDF

With knowledge of the entropy powers, the question now is how we can reconstruct the information distribution g(x). The inner workings of this will now be explicitly illustrated with the (generalized) Gram-Charlier A expansion. However, other—often more efficient methods—are also available [91]. Let $\kappa_{n}$ be cumulants obtained from entropy powers and let G(x) be some reference PDF whose cumulants are $\gamma_{k}$. The information PDF g(x) can be then written as [91]

(41) $$\begin{array}{c} \;g\left(x\right)\;=\;\exp\left[\sum_{k=1}^{\infty }\left(\kappa_{k}-\gamma_{k}\right){\left(-1\right)}^{k}\frac{\left(d^{k}/dx^{k}\right)}{k!}\right]G\left(x\right)\;. \end{array}$$

With hindsight, we choose the reference PDF G(x) to be a shifted gamma PDF, i.e.,

(42) $$\begin{array}{ccc} \;G(x)\; & \equiv & \;G\left(x|a,\alpha ,\beta \right)\;=\;\frac{e^{-(x-a)/\beta }{\left(x-a\right)}^{\alpha -1}}{\beta^{\alpha }\Gamma \left[\alpha \right]}\;, \end{array}$$

with a<x<∞,β>0,α>0. In doing so, we have implicitly assumed that the F(y) PDF is in the first approximation equimeasurable with the Gaussian PDF. To reach a corresponding matching, we should choose $a=\log_{2}\left(2\pi \sigma^{2}\right)/2$, α=1/2 and $\beta =\log_{2}e$. Using the fact that [95]

(43) $$\begin{array}{c} {\left(\beta \right)}^{k+1/2}\;\;\frac{d^{k}\;\;G\left(x|a,1/2,\beta \right)}{k!\;\;dx^{k}}\;=\;{\left(\frac{x-a}{\beta }\right)}^{-k}L_{k}^{\left(-1/2-k\right)}\left(\frac{x-a}{\beta }\right)G\left(x|a,1/2,\beta \right)\;, \end{array}$$

(where $L_{k}^{\delta }$ is an associated Laguerre polynomial of order *k* with parameter δ) and given that $\kappa_{1}=\gamma_{1}=\alpha \beta +a=\log_{2}\left(2\pi \sigma^{2}e\right)/2$, and $\gamma_{k}=\Gamma \left(k\right)\alpha \beta^{k}={\left(\log_{2}e\right)}^{k}/2$ for k>1 we can write (41) as

(44) $$\begin{array}{ccc} \;g(x) & = & G\left(x|a,1/2,\beta \right)\left[1\;+\;\frac{\left(\kappa_{2}-\gamma_{2}\right)}{\beta^{1/2}{\left(x-a\right)}^{2}}\;L_{2}^{\left(-5/2\right)}\left(\frac{x-a}{\beta }\right)\right.\\ & - & \left.\frac{\left(\kappa_{3}-\gamma_{3}\right)}{\beta^{1/2}{\left(x-a\right)}^{3}}\;L_{3}^{\left(-7/2\right)}\left(\frac{x-a}{\beta }\right)\;+\;\cdots \right]. \end{array}$$

If needed, one can use a relationship between the moments and the cumulants (Faà di Bruno’s formula [94]) to recast the expansion (44) into more familiar language. For the Gram–Charlier A expansion, various formal convergence criteria exist (see, e.g., [91]). In particular, the expansion for nearly Gaussian equimeasurable PDFs F(y) converges quite rapidly and the series can be truncated fairly quickly. Since in this case one needs fewer $\kappa_{k}$’s in order to determine the information PDF g(x), only EPs in the small neighborhood of the index 1 will be needed. On the other hand, the further the F(y) is from Gaussian (e.g., heavy-tailed PDFs), the higher the orders of $\kappa_{k}$ are required to determine g(x), and hence a wider neighborhood of the index 1 will be needed for EPs.

## 6. Example—Reconstruction Theorem and (Un)Balanced Cat State

**Figure 1:**
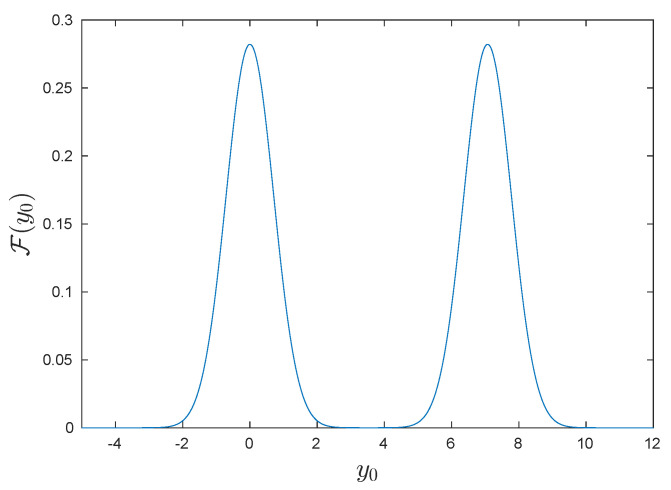
Probability distribution function of a balanced cat state (BCS) for the quantum mechanical state’s position-like quadrature variable with α=5. This clearly displays an overall non-Gaussian structure; however, as this is a piecewise rearrangement of a Gaussian PDF for all α, we have that $N_{1-p}=\sigma^{2}$ for all *p* and α.

We now demonstrate an example of the reconstruction in the context of a quantum system. Specifically, we consider cat states that are often considered in the foundations of quantum physics as well as in various applications, including solid state physics [96] and quantum metrology [97]. The form of the state we consider is ∣ψ〉=N(∣0〉+ν∣α/ν〉), where $N={\left[1+2\nu \exp\left(-\alpha^{2}/2\nu^{2}\right)+\nu^{2}\right]}^{-1/2}$ is the normalization factor, ∣0〉 is the vacuum state, ν∈R a weighting factor, and ∣α〉 is the coherent state given by

(45) $$\begin{array}{c} |\;\alpha \rangle \;=\;e^{-\alpha^{2}/2}\sum_{n=0}^{\infty }\frac{\alpha^{n}}{\sqrt{n!}}\;\;|\;n\rangle \;, \end{array}$$

(taking α∈R). For ν=1, we refer to the state as a *balanced cat state* (BCS) and for ν≠1, as an *unbalanced cat state* (UCS). Changing the basis of ∣ψ〉 to the eigenstates of the general quadrature operator

(46) $$\begin{array}{c} {\hat{Y}}_{\theta }\;=\;\frac{1}{\sqrt{2}}\left(\hat{a}e^{-i\theta }+{\hat{a}}^{†}e^{i\theta }\right)\;, \end{array}$$

where $\hat{a}$ and ${\hat{a}}^{†}$ are the creation and annihilation operators of the electromagnetic field, we find the PDF for the general quadrature variable $y_{\theta }$ to be

(47) $$\begin{array}{c} F\left(y_{\theta }\right)\;=\;N^{2}\pi^{-\frac{1}{2}}e^{-y_{\theta }^{2}}\;\;{\left|1+\nu \exp\left[-\frac{\alpha^{2}}{\nu^{2}2}\left(1+e^{2i\theta }-2\sqrt{2}e^{i\theta }\frac{\nu }{\alpha }y_{\theta }\right)\right]\right|}^{2}\;, \end{array}$$

where N is the normalization constant. Setting θ=0 and ν=1 returns the PDF of the BCS for the position-like variable $y_{0}$. With this, the Rényi EPs $N_{1-p}\left(\chi \right)$ are calculated and found to be constant across varying *p*. This is because $F(y_{0})$ for the BCS is in fact a piecewise rearrangement of a Gaussian PDF (yet has an overall non-Gaussian structure) as depicted in Figure 1, thus $N_{1-p}\left(\chi \right)=\sigma^{2}$ for all *p*, where $\sigma^{2}$ is the variance of the ‘would be Gaussian’. Taking the reference PDF to be G(x)=G(x|a,α,β), with $a=\log_{2}\left(2\pi \sigma^{2}\right)/2$, α=1/2 and $\beta =\log_{2}\left(e\right)$, it is evident that $(\kappa_{k}-\gamma_{k})=0$ for all k≥1, and from the Gram–Charlier A series (41), a perfect matching in the reconstruction is achieved. Furthermore, it can be shown that the variance of (47) increases with α, i.e., the variance increases as the peaks of the PDF diverge, which is in stark contrast to the Rényi EPs which remain constant for increasing α. This reveals the shortcomings of variance as a measure of uncertainty for non-Gaussian PDFs.

The peaks, located at $F\left(y_{\theta }\right)=2^{-a_{j}^{+}}$, where *j* is an index labelling the distinct peaks, give rise to sharp singularities in the target g(x). With regard to the BCS position PDF, distributions of the conjugate parameter $F(y_{\pi /2})$ distinguish $F(y_{0})$ from its equimeasurable Gaussian PDF and hence the Rényi EPs also distinguish the different cases. The number of available cumulants *k* is computationally limited, but, as this grows, information about the singularities will be recovered in the reconstruction. In the following, we show how the tail convergence and location of a singularity for g(x) can be reconstructed using k=5.

**Figure 2:**
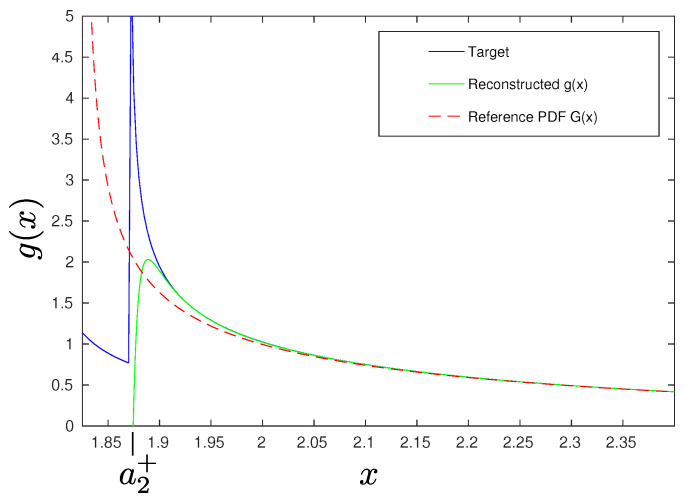
Reconstructed information distribution of an unbalanced cat state with ν=0.97 and α=10. The Edgeworth expansion has been used here to order $n^{-3/2}$ requiring control of the first five REPs. Good convergence of the tail behavior is evident as well as the location of the singularity corresponding to the second peak; $a_{2}^{+}$ corresponds to the value of *x* at the point of intersection with the second (lower) peak of $F(y_{0})$.

We consider the case of a UCS with ν=0.97, α=10 and we take θ=0 in Equation (47) to find the PDF in the $y_{0}$ quadrature which is non-Gaussian for all piecewise rearrangements. As such, all REPs $N_{1-p}$ vary with *p* and consequently all cumulants $\kappa_{k}$ carry information on g(x). Here, we choose to reconstruct the UCS information distribution by means of the Edgeworth series [91] so that

(48) $$\;g\left(x\right)=\exp\left[n\sum_{j=2}^{\infty }\left(\kappa_{j}-\gamma_{j}\right)\frac{{\left(-1\right)}^{j}}{j!}\frac{d^{j}}{dx^{j}}n^{-j/2}\right]G\left(x\right),$$

where the reference PDF G(x) is again the shifted gamma distribution. Using the Edgeworth series, the information PDF is approximated by expanding in orders of *n*, which has the advantage over the Gram–Charlier A expansion discussed above of bounding the errors of the approximation. For the particular UCS of interest, expanding to order $n^{-3/2}$ reveals convergence toward the analytic form of the information PDF shown as the target line in Figure 2. This shows that, for a given characteristic resolution, control over the first five Rényi EPs can be enough for a useful information scan of a quantum state with an underlying non-Gaussian PDF. In the example shown in Figure 2, we see that the information scan accurately predicts the tail behavior as well as the location of the singularity, which corresponds to the second (lower) peak of $F(y_{0})$.

## 7. Entropy Powers Based on Tsallis Entropy

Let us now briefly comment on the entropy powers associated with yet another important differential entropy, namely *Tsallis differential entropy*, which is defined as [47]

(49) $$\begin{array}{c} S_{q}\left(F\right)\;=\;\frac{1}{\left(1-q\right)}\left[\int_{R^{D}}\left(F^{q}\left(x\right)-F\left(x\right)\right)dx\right]\;, \end{array}$$

where, as before, the PDF F(x) is associated with a random vector $\left\{X_{i}\right\}$ in $R^{D}$.

Similarly to the RE case, the Tsallis entropy power $N_{p}^{T}\left(X\right)$ is defined as the solution of the equation

(50) $$\begin{array}{c} S_{q}\left(X\right)\;=\;S_{q}^{T}\left(\sqrt{N_{q}^{T}\left(X\right)}\cdot Z^{{}_{G}}\right)\;. \end{array}$$

The ensuing entropy power has not been studied in the literature yet, but it can be easily derived by observing that the following scaling property for differential Tsallis entropy holds, namely

(51) $$\begin{array}{c} S_{q}\left(aX\right)\;=\;S_{q}\left(X\right)\;{⊕}_{q}\;\ln_{q}{\left|a\right|}^{D}\;, \end{array}$$

where a∈R and the *q*-deformed sum and logarithm are defined as [11]: $x{⊕}_{q}y=x+y+\left(1-q\right)xy$ and $\ln_{q}x=\left(x^{1-q}-1\right)/\left(1-q\right)$, respectively. Relation (51) results from the following chain of identities:

(52) $$\begin{array}{ccc} S_{q}\left(aX\right)\; & = & \;\frac{1}{1-q}\left[\int_{R^{D}}d^{D}y{\left(\int_{R^{D}}d^{D}x\;\;\delta \left(y-ax\right)F\left(x\right)\right)}^{\;q}-1\right]\\ & = & \frac{1}{1-q}\left[{\left|a\right|}^{D(1-q)}\;\;\int_{R^{D}}d^{D}y\;\;F^{q}\left(y\right)-1\right]\\ & = & {\;|a|}^{D(1-q)}\;\;\left(S_{q}\left(X\right)\;+\;\frac{1}{1-q}\right)\;-\;\frac{1}{1-q}\\ & = & {\;|a|}^{D(1-q)}\;\;S_{q}\left(X\right)\;+\;\ln_{q}{\left|a\right|}^{D}\\ & = & \;\left[\left(1-q\right)\ln_{q}{\left|a\right|}^{D}\;+\;1\right]S_{q}\left(X\right)\;+\;\ln_{q}{\left|a\right|}^{D}\\ & = & S_{q}\left(X\right)\;{⊕}_{q}\;\ln_{q}{\left|a\right|}^{D}\;. \end{array}$$

We can further use the simple fact that

(53) $$\begin{array}{c} S_{q}\left(Z_{G}\right)\;=\;\ln_{q}{\left(2\pi q^{q^{\prime }/q}\right)}^{D/2}\;. \end{array}$$

Here, *q* and $q^{\prime }$ satisfy $1/q+1/q^{\prime }=1$ with $q\in R^{+}$. By combining (50), (51), and (53), we get

(54) $$\begin{array}{c} S_{q}\left(X\right)\;=\;\ln_{q}{\left(2\pi q^{q^{\prime }/q}\right)}^{D/2}\;{⊕}_{q}\;\ln_{q}{\left(N_{q}^{T}\right)}^{D}/2\;=\;\ln_{q}{\left(2\pi q^{q^{\prime }/q}N_{q}^{T}\right)}^{D/2}\;, \end{array}$$

where we have used the sum rule from the *q*-deformed calculus: $\ln_{q}x{⊕}_{q}\ln_{q}y=\ln_{q}xy$. Equation (54) can be resolved for $N_{p}^{T}$ by employing the *q*-exponential, i.e., $e_{q}^{x}={\left[1+\left(1-q\right)x\right]}^{1/(1-q)}$, which (among others) satisfies the relation $e_{q}^{\ln_{q}x}=\ln_{q}\left(e_{q}^{x}\right)=x$. With this, we have

(55) $$\begin{array}{c} N_{q}^{T}\left(X\right)\;=\;\frac{1}{2\pi }q^{-q^{\prime }/q}{\left[\exp_{q}\left(S_{q}\left(X\right)\right)\right]}^{2/D}\;=\;\frac{1}{2\pi }q^{-q^{\prime }/q}\exp_{1-(1-q)D/2}\left(\frac{2}{D}\;\;S_{q}\left(X\right)\right)\;. \end{array}$$

In addition, when $q\to 1_{+}$, one has

(56) $$\begin{array}{c} \lim_{q\to 1}N_{q}^{T}\left(X\right)\;=\;\frac{1}{2\pi e}\exp\left(\frac{2}{D}H\left(X\right)\right)\;=\;N\left(X\right)\;, \end{array}$$

where N(X) is the conventional Shannon entropy power and H(X) is the Shannon entropy [5].

In connection with Tsallis EP, we might notice one interesting fact, namely by starting from Rényi’s EP (considering RE in nats), we can write

(57) $$\begin{array}{ccc} N_{q}\left(X\right)\; & = & \;\frac{1}{2\pi }q^{-q^{\prime }/q}\exp\left(\frac{2}{D}\;\;I_{q}\left(X\right)\right)\;=\;\frac{1}{2\pi }q^{-q^{\prime }/q}{\left(\int d^{D}x\;\;F^{q}\left(x\right)\right)}^{2/(D(1-q))}\\ & = & \;\frac{1}{2\pi }q^{-q^{\prime }/q}{\left[e_{q}^{S_{q}^{T}\left(X\right)}\right]}^{2/D}\;=\;N_{q}^{T}\left(X\right)\;. \end{array}$$

Here, we have used a simple identity

(58) $$\begin{array}{c} {\left(\int d^{D}x\;\;F^{q}\left(x\right)\right)}^{1/\left(1-q\right)}\;=\;{\left[\left(1-q\right)S_{q}^{T}\left(X\right)\;+\;1\right]}^{1/(1-q)}\;=\;e_{q}^{S_{q}^{T}\left(X\right)}\;. \end{array}$$

Thus, we have obtained that Rényi and Tsallis EPs coincide with each other. In particular, Rényi’s EPI (22) can be equivalently written in the form

(59) $$\begin{array}{c} N_{p/2}^{T}\left(X\right)N_{q/2}^{T}\left(Y\right)\;\geq \;\frac{1}{16\pi^{2}}\;. \end{array}$$

Similarly, we could rephrase the generalized Stam inequality (21) and generalized isoperimetric inequality (14) in terms of Tsallis EPs. Though such inequalities are quite interesting from a mathematical point of view, it is not yet clear how they could be practically utilized in the estimation theory as there is no obvious operational meaning associated with Tsallis entropy (e.g., there is no coding theorem for Tsallis entropy). On the other hand, Tsallis entropy is an important concept in the description of entanglement [98]. For instance, Tsallis entropy of order 2 (also known as linear entropy) directly quantifies state purity [63].

## 8. Conclusions

In the first part of this paper, we have introduced the notion of Rényi’s EP. With quantum metrology applications in mind, we carried out our discussion in the framework of estimation theory. In doing so, we have generalized the notion of Fisher information (FI) by using a Rényi entropy version of De Bruijn’s identity. The key role of the escort distribution in this context was highlighted. With Rényi’s EP at hand, we proved the RE-based isoperimetric and Stam inequalities. We have further clarified the role of Rényi’s EP by deriving (through the generalized Stam inequality) a one-parameter family of Rényi EP-based quantum mechanical uncertainty relations. Conventional variance-based URs of Robertson-Schrödinger and Shannon differential entropy-based URs of Hirschman or Białynicki-Birula naturally appear as special cases in this hierarchy of URs. Interestingly, we found that the Tsallis entropy-based EP coincided with Rényi’s EP provided that the order is the same. This might open quite a new, hitherto unknown role for Tsallis entropy in estimation theory.

The second part of the paper was devoted to developing the application of Rényi’s EP for extracting quantum states from incomplete data. This is of particular interest in various quantum metrology protocols. To that end, we introduced the concepts of information distribution and showed how cumulants of the information distribution can be obtained from knowledge of EPs of various orders. With cumulants thus obtained, one can reconstruct the underlying information distribution in a process which we call an information scan. A numerical implementation of this reconstruction procedure was technically realized via Gram-Charlier A and Edgeworth expansion. For an explicit illustration of the information scan, we used the non-Gaussian quantum states—(un)balanced cat states. In this case, it was found that control of the first five significant Rényi EPs gave enough information for a meaningful reconstruction of the information PDF and brought about non-trivial information on the original balanced cat state PDF, such as asymptotic tail behavior or the heights of the peaks.

Finally, let us stress one more point. Rényi EP-based quantum mechanical uncertainty relations (26) basically represent a one-parameter class of inequalities that constrain higher-order cumulants of state distributions for conjugate observables [13]. In connection with this, the following two questions are in order. Assuming one is able to control Rényi EPs of various orders: (i) how do such Rényi EPs constrain the underlying state distribution and (ii) how do the ensuing REPURs restrict the state distributions of conjugate observables? The first question was tackled in this paper in terms of the information distribution and reconstruction theorem. The second question is more intriguing and has not yet been properly addressed. Work along these lines is presently under investigation.

## Data Availability

Not applicable.

## Abbreviations

The following abbreviations are used in this manuscript:

ITE	Information-theoretic entropy
UR	Uncertainty relation
RE	Rényi entropy
TE	Tsallis entropy
REPUR	Rényi entropy-power-based quantum uncertainty relation
QM	Quantum mechanics
EP	Entropy power
FI	Fisher information
PDF	Probability density function
EPI	Entropy power inequality
REP	Rényi entropy power
BCS	Balanced cat state
UCS	Unbalanced cat state

## Appendix A

Here, we provide an intuitive proof of the generalized De Bruijn identity.

*Generalized De Bruijn identity I:* By denoting the PDF associated with a random vector $\left\{X_{i}\right\}$ as F(x) and the noise PDF as G(z), we might write the LHS of (7) as

(A1) $$\begin{array}{ccc} & & \;\frac{d}{d\epsilon }I_{q}\left(X+\sqrt{\epsilon }\;\;Z\right){|}_{\epsilon =0}\\ & = & {\left.\frac{1}{1-q}\frac{d}{d\epsilon }\log\left[\int_{R^{D}}dy{\left(\int_{R^{D}}dx\int_{R^{D}}dz\;\;\delta^{\left(D\right)}\left(y-(x+\sqrt{\epsilon }z)\right)F\left(x\right)G\left(z\right)\right)}^{\;\;q}\;\;\right]\right|}_{\epsilon =0}\\ & = & {\left.\frac{1}{1-q}\frac{d}{d\epsilon }\log\left[\int_{R^{D}}dy{\left(\int_{R^{D}}dz\;\;F\left(y-\sqrt{\epsilon }z\right)G\left(z\right)\right)}^{\;\;q}\;\;\right]\right|}_{\epsilon =0}\\ & = & \left.\frac{1}{1-q}\frac{d}{d\epsilon }\log\left\{\int_{R^{D}}dy\left[\int_{R^{D}}dz\;\;\left(F\left(y\right)-\sqrt{\epsilon }z_{i}\nabla_{i}F\left(y\right)\right.\right.\right.\right.\\ & + & {\left.\left.{\left.\left.\frac{1}{2}\epsilon z_{i}z_{j}\nabla_{i}\nabla_{j}F\left(y\right)\;+\;O\left(\epsilon^{3/2}\right)\right)G\left(z\right)\right]}^{\;q}\;\;\right\}\right|}_{\epsilon =0}\\ & = & \frac{q}{1-q}\left[\int_{R^{D}}dy\;\;\rho_{q}\left(y\right)\Sigma_{ij}\frac{\nabla_{i}\nabla_{j}F\left(y\right)}{2F(y)}\right]\;=\;\frac{q}{2}\left[\int_{R^{D}}dy\;\;\rho_{q}\left(y\right)\Sigma_{ij}V_{i}\left(y\right)V_{j}\left(y\right)\right]\\ & = & \frac{q}{2}\;\;\mathrm{Tr}\left[{\mathrm{cov}}_{q}\left(V\right)\Sigma \right]\;=\;\frac{1}{2q}\;\;\mathrm{Tr}\left[{\mathrm{cov}}_{q}\left(V_{q}\right)\Sigma \right]\;=\;\frac{1}{2q}\;\;\mathrm{Tr}\left(J_{q}\Sigma \right)\;. \end{array}$$

It should be noted that our manipulations make sense only for any q>0, as only in these cases are RE and escort distributions well defined. The right-hand-side of (A1) can also be equivalently written as

(A2) $$\begin{array}{ccc} & & \;\frac{1}{2q}E_{q}\left\{\left[{\left(V_{q}\right)}_{i}-E_{q}\left({\left(V_{q}\right)}_{i}\right)\right]\Sigma_{ij}\left[{\left(V_{q}\right)}_{j}-E_{q}\left({\left(V_{q}\right)}_{j}\right)\right)]\right\}\;,\\ & & \;=\;\frac{1}{2q}E\left\{\left[(Z_{i}-E\left(Z_{i}\right)\right]{\left(J_{q}\right)}_{ij}\left(X\right)\left[Z_{j}-E\left(Z_{j}\right)\right]\right\}\;, \end{array}$$

where the mean $E_{q}\left\{\ldots \right\}$ is performed with respect to the escort distribution $\rho_{q}$, while E with respect to G distribution.

We note in passing that the conventional De Bruijn’s identity (6) emerges as a special case when q→1. For the Gaussian noise vector, we can generalize the previous derivation in the following way:

*Generalized De Bruijn’s identity II:* Let $\left\{X_{i}\right\}$ be a random vector in $R^{D}$ with the PDF F(x) and let $\left\{Z_{i}\right\}$ be an independent Gaussian noise vector with the zero mean and covariance matrix $\Sigma =\mathrm{cov}(Z^{{}_{G}})$, then,

(A3) $$\begin{array}{ccc} \frac{d}{d\Sigma_{ij}}I_{q}\left(X+Z^{{}_{G}}\right){|}_{{}_{\Sigma =0}} & = & \frac{q}{1-q}\left[\int_{R^{D}}\;\;dy\;\;\rho_{q}\left(y\right)\frac{\nabla_{i}\nabla_{j}F\left(y\right)}{2F(y)}\right]\\ & = & \frac{1}{2q}\left[\int_{R^{D}}\;\;dy\;\;\rho_{q}\left(y\right){\left(V_{q}\right)}_{i}{\left(V_{q}\right)}_{j}\right]\;=\;\frac{1}{2q}\;\;{\left(J_{q}\right)}_{ij}\;. \end{array}$$

The right-hand-side is equivalent to

(A4) $$\begin{array}{c} \frac{1}{2q}E_{q}\left\{\left[{\left(V_{q}\right)}_{i}-E_{q}\left({\left(V_{q}\right)}_{i}\right)\right]\left[{\left(V_{q}\right)}_{j}-E_{q}\left({\left(V_{q}\right)}_{j}\right)\right)]\right\}\;. \end{array}$$

To prove the identity (A3), we might follow the same line of reasoning as in (A1). The only difference is that, while in (A1) we had a small parameter ϵ in which one could expand to all orders of correlation functions and easily perform differentiation and limit ϵ→0 for any noise distribution (with zero mean), the same procedure can not be done in the present context for a generic noise distribution. In fact, only the Gaussian distribution has the property that the higher-order correlation functions and their derivatives with respect to $\Sigma_{ij}$ are small when Σ is small. The latter is a consequence of the Marcinkiewicz theorem [99].

## Appendix B

Here, we prove the *Generalized isoperimetric inequality* from Section 2. The starting point is the *entropy-power inequality* (EPI) [64]: Let $X_{1}$ and $X_{2}$ be two independent continuous random vectors in $R^{D}$ with probability densities $F^{\left(1\right)}\in \ell^{q}\left(R^{D}\right)$ and $F^{\left(2\right)}\in \ell^{p}\left(R^{D}\right)$, respectively. Suppose further that λ∈(0,1) and r>1, and let

(A5) $$\begin{array}{c} q\;=\;\frac{r}{(1-\lambda )+\lambda r}\;,\;\;\;\;p=\frac{r}{\lambda +(1-\lambda )r}\;, \end{array}$$

then the following inequality holds:

(A6) $$\begin{array}{c} N_{r}\left(X_{1}+X_{2}\right)\;\geq \;{\left(\frac{N_{q}\left(X_{1}\right)}{1-\lambda }\right)}^{1-\lambda }{\left(\frac{N_{p}\left(X_{2}\right)}{\lambda }\right)}^{\lambda }. \end{array}$$

Let us now consider a Gaussian noise vector $Z^{{}_{G}}$ (independent of X) with zero mean and covariance matrix Σ. Within this setting, we can write the following EPIs:

(A7) $$\begin{array}{c} N_{r}\left(X+Z^{{}_{G}}\right)\;\geq \;\epsilon^{\lambda }{\left(\frac{1}{1-\lambda }\right)}^{1-\lambda }{\left(\frac{1}{\lambda }\right)}^{\lambda }{\left[N_{q}\left(X\right)\right]}^{1-\lambda }\;, \end{array}$$

(A8) $$\begin{array}{c} N_{r}\left(X+Z^{{}_{G}}\right)\;\geq \;\epsilon^{1-\lambda }{\left(\frac{1}{1-\lambda }\right)}^{1-\lambda }{\left(\frac{1}{\lambda }\right)}^{\lambda }{\left[N_{p}\left(X\right)\right]}^{\lambda }\;, \end{array}$$

with $\epsilon \equiv \det{\left(\Sigma \right)}^{1/D}$. Here, we have used the simple fact that $N_{r}\left(Z^{{}_{G}}\right)=\det{\left(\Sigma \right)}^{1/D}$, irrespective of the value of *r*.

Let us now fix *r* and maximize the RHS of inequality (A7) with respect to λ and *q* provided we keep the constraint condition (A5). This yields the condition extremum

(A9) $$\begin{array}{c} \lambda \;=\;\frac{\epsilon }{N_{q}\left(X\right)}\;\;\exp\left[q\left(1-q\right)\frac{d\log N_{q}\left(X\right)}{dq}\right]\;+\;O\left(\epsilon^{2}\right)\;. \end{array}$$

With this, *q* turns out to be

(A10) $$\begin{array}{c} q\;=\;r\;+\;\frac{\epsilon (1-r)r}{N_{r}\left(X\right)}\;\;\exp\left[\left(1-r\right)r\frac{d\log N_{r}\left(X\right)}{dr}\right]\;+\;O\left(\epsilon^{2}\right)\;, \end{array}$$

which in the limit ϵ→0 reduces to q=r≥1. The latter implies that p=1. The result (A10) implies that the RHS of (A7) reads

(A11) $$\begin{array}{c} N_{q}\left(X\right)\;+\;\epsilon \;\;\exp\left[\left(1-r\right)r\frac{d\log N_{r}\left(X\right)}{dr}\right]\left[1-\left(1-r\right)r\frac{d\log N_{r}\left(X\right)}{dr}\right]\;+\;O\left(\epsilon^{2}\right)\;. \end{array}$$

Should we have started with the *p* index, we would arrive at an analogous conclusion. To proceed, we stick, without loss of generality, to the inequality (A7). This implies that

(A12) $$\begin{array}{ccc} N_{r}\left(X+Z^{{}_{G}}\right) & \geq & \;N_{q}\left(X\right)\\ & + & \;\epsilon \;\;\exp\left[\left(1-r\right)r\frac{d\log N_{r}\left(X\right)}{dr}\right]\left[1-\left(1-r\right)r\frac{d\log N_{r}\left(X\right)}{dr}\right]\\ & + & \;+\;O\left(\epsilon^{2}\right)\\ & = & \;N_{r}\left(X\right)\;+\;\left[N_{q}\left(X\right)-N_{r}\left(X\right)\right]\\ & + & \;\epsilon \;\;\exp\left[\left(1-r\right)r\frac{d\log N_{r}\left(X\right)}{dr}\right]\left[1-\left(1-r\right)r\frac{d\log N_{r}\left(X\right)}{dr}\right]\\ & + & \;O\left(\epsilon^{2}\right)\\ & \geq & \;N_{r}\left(X\right)\;+\;\epsilon \;\;\exp\left[\left(1-r\right)r\frac{d\log N_{r}\left(X\right)}{dr}\right]\;+\;O\left(\epsilon^{2}\right)\;. \end{array}$$

To proceed, we employ the identity $\log N_{r}\left(X\right)=2/D\left[I_{r}\left(X\right)-I_{r}\left(Z_{1\;I}^{{}_{G}}\right)\right]$ with $Z_{1\;I}^{{}_{G}}$ representing a Gaussian random vector with zero mean and *unit* covariance matrix, and the fact that $I_{r}$ is monotonously decreasing function of *r*, i.e., $dI_{r}/dr\leq 0$ (see, e.g., Ref. [78]). With this, we have

(A13) $$\begin{array}{ccc} \exp\left[\left(1-r\right)r\frac{d\log N_{r}\left(X\right)}{dr}\right]\; & \geq & \;\exp\left[\frac{2(r-1)r}{D}\;\;\frac{dI_{r}\left(Z_{1\;I}^{{}_{G}}\right)}{dr}\right]\\ & = & \;\exp\left[\left(r-1\right)r\frac{d}{dr}\;\left(\frac{1}{r-1}\log r\right)\right]\\ & = & \;er^{r/(r-1)}\;\geq \;\frac{e^{2}}{r}\;. \end{array}$$

Consequently, Equation (A12) can be rewritten as

(A14) $$\begin{array}{ccc} & & \;\frac{N_{r}\left(X+Z^{{}_{G}}\right)\;-\;N_{q}\left(X\right)}{\Sigma_{ij}}\;\geq \;\frac{\epsilon }{\Sigma_{ij}}\frac{e^{2}}{r}\;+\;O\left(\epsilon^{2}/\Sigma_{ij}\right)\;. \end{array}$$

At this stage, we are interested in the $\Sigma_{ij}\to 0$ limit. In order to find the ensuing leading order behavior of $\epsilon /\Sigma_{ij}$, we can use L’Hospital’s rule, namely

(A15) $$\begin{array}{c} \frac{\epsilon }{\Sigma_{ij}}\;=\;\frac{d\epsilon }{d\Sigma_{ij}}\;=\;\frac{d}{d\Sigma_{ij}}\exp\left[\frac{1}{D}\mathrm{Tr}\left(\log\Sigma \right)\right]\;=\;\frac{\epsilon }{D}{\left(\Sigma^{-1}\right)}_{ij}\;. \end{array}$$

Now, we neglect the sub-leading term of order $O(\epsilon^{2}/\Sigma_{ij})$ in (A14) and take $\det{\left(\ldots \right)}^{1/D}$ on both sides. This gives

(A16) $$\begin{array}{c} {\left.\det{\left(\frac{dN_{r}\left(X+Z^{{}_{G}}\right)}{d\Sigma_{ij}}\right)}^{1/D}\right|}_{\Sigma =0}\;=\;\frac{1}{rD}N_{r}\left(X\right){\left[\det\left(J_{r}\left(X\right)\right)\right]}^{1/D}\;\geq \;\frac{e^{2}}{rD}\;\geq \;\frac{1}{rD}\;, \end{array}$$

or equivalently

(A17) $$\begin{array}{c} N_{r}\left(X\right){\left[\det\left(J_{r}\left(X\right)\right)\right]}^{1/D}\;\geq \;1\;. \end{array}$$

At this stage, we can use the inequality of arithmetic and geometric means to write (note that $J_{r}={\mathrm{cov}}_{r}\left(V_{r}\right)$ is a positive semi-definite matrix)

(A18) $$\begin{array}{c} \frac{1}{D}\mathrm{Tr}\left(J_{r}\left(X\right)\right)\;\geq \;{\left[\det\left(J_{r}\left(X\right)\right)\right]}^{1/D}\;. \end{array}$$

Consequently, we have

(A19) $$\begin{array}{c} \frac{1}{D}N_{r}\left(X\right)\mathrm{Tr}\left(J_{r}\left(X\right)\right)\;=\;\frac{1}{D}N_{r}\left(X\right)J_{r}\left(X\right)\;\geq \;N_{r}\left(X\right){\left[\det\left(J_{r}\left(X\right)\right)\right]}^{1/D}\;\geq \;1\;, \end{array}$$

as stated in Equation (14).

## Appendix C

In this appendix, we prove the *Generalized Stam inequality* from Section 2. We start with the defining relation (13), i.e.,

(A20) $$\begin{array}{c} N_{q}\left(Y\right)\;=\;\frac{1}{2\pi }q^{1/(1-q)}{\left|\;|G|\;\right|}_{q}^{2q/[(1-q)D]}\;, \end{array}$$

and consider q∈[1/2,1] so that q/(1−q)>0. For the *q*-norm, we can write

(A21) $$\begin{array}{c} {\left|\;|G|\;\right|}_{q}\;=\;{\left(\int_{R^{D}}dy\;\;{\left|\psi_{{}_{G}}\left(y\right)\right|}^{2q}\right)}^{1/q}\;=\;|\;|\psi_{{}_{G}}{\left|\;\right|}_{2q}^{2}\;\geq \;|\;|{\hat{\psi }}_{{}_{G}}{\left|\;\right|}_{2r}^{2}\;=\;|\;|\psi_{{}_{F}}{\left|\;\right|}_{2r}^{2}\;={\;|\;|F|\;|}_{r}\;. \end{array}$$

Here, 2r and 2q are Hölder conjugates so that r∈[1,∞]. The inequality employed is due to the Hausdorff–Young inequality (which in turn is a simple consequence of the Hölder inequality [64]). We further have

(A22) $$\begin{array}{ccc} {\left|\;|F|\;\right|}_{r} & = & {\left(\int_{R^{D}}dx\;\;{\left|\psi_{{}_{F}}\left(x\right)\right|}^{2r}\right)}^{1/r}\geq {\left|\int_{R^{D}}dx\;\;{\left|\psi_{{}_{F}}\left(x\right)\right|}^{2r}\frac{\nabla_{i}\nabla_{i}\;\;e^{ia\cdot x}}{a_{i}^{2}}\right|}^{1/r}\\ & = & {\left|r\int_{R^{D}}dx\;\;\left[\left(r-1\right)\rho_{r}\left(x\right)V_{i}\left(x\right)V_{i}\left(x\right)+\rho_{r}\left(x\right)\frac{\nabla_{i}\nabla_{i}\;\;F\left(x\right)}{F(x)}\right]e^{ia\cdot x}\right|}^{1/r}\;\\ & & \times \;\frac{{\left(\int_{R^{D}}dx\;\;{\left|\psi_{{}_{F}}\left(x\right)\right|}^{2r}\right)}^{1/r}}{a_{i}^{2/r}}\\ & \geq & {\left|r\int_{R^{D}}dx\;\;\left[\left(r-1\right)\rho_{r}\left(x\right)V_{i}\left(x\right)V_{i}\left(x\right)+\rho_{r}\left(x\right)\frac{\nabla_{i}\nabla_{i}\;\;F\left(x\right)}{F(x)}\right]\cos\left(a\cdot x\right)\right|}^{1/r}\;\\ & & \times \;\frac{{\left(\int_{R^{D}}dx\;\;{\left|\psi_{{}_{F}}\left(x\right)\right|}^{2r}\right)}^{1/r}}{a_{i}^{2/r}}\\ & \geq & {\left|r\int_{V_{D}}dx\;\;\rho_{r}\left(x\right)\frac{\nabla_{i}\nabla_{i}\;\;F\left(x\right)}{F(x)}\;\;\cos\left(a\cdot x\right)\right|}^{1/r}\;\frac{{\left(\int_{R^{D}}dx\;\;{\left|\psi_{{}_{F}}\left(x\right)\right|}^{2r}\right)}^{1/r}}{a_{i}^{2/r}}\;, \end{array}$$

where $a\in R^{D}$ is an arbitrary x-independent vector, $\nabla_{i}\equiv \partial /\partial x_{i}$ and $V_{D}$ denotes a regularized volume of $R^{D}$—*D*-dimensional ball of a very large (but finite) radius *R*. In the first line of (A22), we have employed the triangle inequality $|E_{r}\left(e^{ia\cdot x}\right)|\leq 1$ (with equality if and only if a=0), namely

(A23) $$\begin{array}{c} \left|\int_{R^{D}}dx\;\;{\left|\psi_{{}_{F}}\left(x\right)\right|}^{2r}e^{ia\cdot x}\right|\;=\;\left|\int_{R^{D}}dx\;\;\rho_{r}\left(x\right)\;\;e^{ia\cdot x}\right|\int_{R^{D}}dx\;\;|\psi_{{}_{F}}{\left(x\right)|}^{2r}\;\leq \;\int_{R^{D}}dx\;\;{\left|\psi_{{}_{F}}\left(x\right)\right|}^{2r}. \end{array}$$

The inequality in the last line holds for $a_{i}=\pi /\left(2R\right)$ (for all *i*), since, in this case, cos(a·x)≥0 for all x from the *D*-dimensional ball. In this case, one may further estimate the integral from below by neglecting the positive integrand $\left(r-1\right)\rho_{r}\left(x\right){\left[V_{i}\left(x\right)\right]}^{2}$.

Note that (A22) implies

(A24) $$\begin{array}{c} \frac{r\left|E_{r}\left[F^{-1}\nabla_{i}\nabla_{i}\;\;F\cos\left(a\cdot x\right)\right]\right|}{a_{i}^{2}}\;\leq \;1\;, \end{array}$$

with equality if and only if a→0 (to see this, one should apply L’Hospital’s rule). Equation (A24) allows for writing

(A25) $$\begin{array}{ccc} {\;|\;|F|\;|}_{r}\; & \geq & \frac{r^{\gamma }{\left|E_{r}\left[F^{-1}\nabla_{i}\nabla_{i}\;\;F\cos\left(a\cdot x\right)\right]\right|}^{\gamma }}{a_{i}^{2\gamma }}{\left(\int_{R^{D}}dx\;\;{\left|\psi_{{}_{F}}\left(x\right)\right|}^{2r}\right)}^{1/r}\\ & \geq & \frac{r^{\gamma }{\left|E_{r}\left[F^{-1}\nabla_{i}\nabla_{i}\;\;F\cos\left(a\cdot x\right)\right]\right|}^{\gamma }}{a_{i}^{2\gamma }}\frac{1}{V_{D}^{1-1/r}}\\ & = & \;\frac{r^{\gamma }{\left|E_{r}\left[F^{-1}\nabla_{i}\nabla_{i}\;\;F\cos\left(a\cdot x\right)\right]\right|}^{\gamma }}{a_{i}^{2\gamma }}\frac{1}{C_{D}^{1-1/r}R^{D-D/r}}\;, \end{array}$$

where γ>0 is some as yet unspecified constant and $C_{D}=\pi^{D/2}/\Gamma \left(D/2+1\right)$. In deriving (A25), we have used the Hölder inequality

(A26) $$\begin{array}{ccc} 1\; & = & \;\left(\int_{R^{D}}dx\;\;1\cdot {\left|\psi_{{}_{F}}\left(x\right)\right|}^{2}\right)\;\leq \;{\left(\int_{R^{D}}dx\;\;1^{r^{\prime }}\right)}^{1/r^{\prime }}{\left(\int_{R^{D}}dx\;\;{\left|\psi_{{}_{F}}\left(x\right)\right|}^{2r}\right)}^{1/r}\\ & = & \;V_{D}^{1-1/r}{\left(\int_{R^{D}}dx\;\;{\left|\psi_{{}_{F}}\left(x\right)\right|}^{2r}\right)}^{1/r}. \end{array}$$

Here, and also in (A22) and (A25), $V_{D}=C_{D}R^{D}$ denotes the regularizated volume of $R^{D}$.

As already mentioned, the best estimate of the inequality (A25) is obtained for a→0. As we have seen, $a_{i}$ goes to zero as π/(2R) which allows for choosing the constant γ so that the denominator in (A25) stays finite in the limit R→∞. This implies that γ=D/2−D/(2r). Consequently, (A25) acquires in the large *R* limit the form

(A27) $$\begin{array}{c} {\left|\;|F|\;\right|}_{r}\;\geq \;\frac{{\left[4\left(r-1\right)/r\right]}^{D/2-D/2r}\;\;{\left[\Gamma \left(D/2+1\right)\right]}^{1-1/r}}{\pi^{3D/2-3D/2r}}\;\;{\left[{\left(J_{r}\right)}_{ii}\left(X\right)\right]}^{D/2-D/2r}\;, \end{array}$$

With this, we can write [see Equations (A20)–(A21)]

(A28) $$\begin{array}{c} N_{q}\left(Y\right)\;\geq \;\frac{1}{{\left(2\pi \right)}^{2}}\;\;q^{1/(1-q)}\;\;\left[{\left(J_{r}\right)}_{ii}\left(X\right)\right]\;\geq \;\frac{1}{16\pi^{2}}\;\;\left[{\left(J_{r}\right)}_{ii}\left(X\right)\right]\;, \end{array}$$

where, in the last inequality, we have used the fact that $q^{1/(1-q)}\geq 1/4$ for q∈[1/2,1] and that ${\left[\Gamma \left(D/2+1\right)\right]}^{2/D}\geq \pi /4$. As a final step, we employ Equations (A18) and (A28) to write

(A29) $$\begin{array}{c} N_{q}\left(Y\right)\;\geq \;\frac{1}{16\pi^{2}D}\;\;\mathrm{Tr}\left(J_{r}\left(X\right)\right)\;\geq \;\frac{1}{16\pi^{2}}\;\;{\left[\det\left(J_{r}\left(X\right)\right)\right]}^{1/D}\;, \end{array}$$

which completes the proof of the generalized Stam’s inequality.
